# Novel Magnetic Mixed Cellulose Acetate Matrix Membranes with Oxygen-Enrichment Potential

**DOI:** 10.3390/membranes12121259

**Published:** 2022-12-13

**Authors:** Norhan Nady, Noha Salem, Mohamed R. Elmarghany, Mohamed S. Salem, Sherif H. Kandil

**Affiliations:** 1 Polymeric Materials Research Department, City of Scientific Research and Technological Applications (SRTA-City), Borg El-Arab City, Alexandria 21934, Egypt; 2 Department of Materials Science, Institute of Graduate Studies and Research, Alexandria University, Alexandria 21526, Egypt; 3 Mechanical Power Engineering Department, Faculty of Engineering, Mansoura University, Mansoura 35516, Egypt

**Keywords:** cellulose acetate, oxygen separation, magnetic mixed matrix, oxygen-enrichment, permanent magnetic membrane

## Abstract

This work presents novel magnetic mixed cellulose-based matrix membranes that combine the advantages of a low-cost common polymer matrix, such as cellulose acetate (CA), and a low-cost magnetic filler. Moreover, the presented magnetic mixed CA matrix membranes were fabricated and used without applying an external magnetic field during either the membrane casting or the separating process. Poly(methylmethacrylate) and lithium chloride were used in order to improve the mechanical properties and porosity of the fabricated membranes. The iron–nickel magnetic alloys used were prepared through a simple chemical reduction method with unique morphologies (Fe_10_Ni_90_—starfish-like and Fe_20_Ni_80_—necklace-like). The novel magnetic mixed CA matrix membranes fabricated were characterized using different analysis techniques, including SEM, EDX, XRD, TGA, and FTIR-ATR analyses. Furthermore, the static water contact angle, membrane thickness, surface roughness, tensile strength, and membrane porosity (using ethanol and water) were determined. In addition, vibrating sample magnetometer (VSM) analysis was conducted and the oxygen transition rate (OTR) was studied. The magnetic mixed CA matrix membrane containing starfish-like Fe_10_Ni_90_ alloy was characterized by high coercivity (109 Oe) and an efficient 1.271 × 10^−5^ cm^3^/(m^2^·s) OTR compared to the blank CA membrane with 19.8 Oe coercivity and no OTR. The effects of the polymeric matrix composition, viscosity, and compatibility with the alloys/fillers used on the structure and performance of the fabricated mixed CA matrix membranes compared to the previously used poly(ethersufone) polymeric matrix are discussed and highlighted. The novel magnetic mixed CA matrix membranes presented have good potential for use in the oxygen-enrichment process.

## 1. Introduction

Mixed matrix membranes (MMMs) are an essential class of organic–inorganic nano-composite membranes that are creating a new horizon for the development of novel materials with the separation properties desired for different applications, such as gas separation [1], water purification [2], biomedical uses [3], fuel-cells [4], and pervaporation [5]. They are formed through the dispersion of inorganic filler particles in the form of micro- or nano-particles into a polymeric matrix. The separation efficiency of MMMs is enhanced through the combination of the simplicity of polymer membrane processing with the strong separation and selection properties of the filler [6]. MMMs have several advantageous properties compared to native polymeric membranes, such as enhanced permselectivity, higher hydrophilicity, high fouling resistance, and high thermal, mechanical, and chemical strength over wider temperature and pH ranges [7,8]. Several techniques are used for the fabrication of MMMs, including the phase inversion method, which involves either evaporation of a volatile solvent from a homogeneous polymer solution or cooling of a casting solution that is homogeneous at elevated temperatures. Another technique is the solution casting method, in which the polymer solution is spread on a flat glass plate with a casting knife, and then the membrane is solidified by immersion in a coagulate bath or by drying [9]. The successful fabrication of MMMs mainly depends on the selection of the polymeric matrix, the filler and its compatibility with the selected polymer, and good dispersion of the filler [10].

Polymeric membrane materials, such as polyimide, polysulfone, and poly(p-phenylene oxide), are the most commonly used polymeric matrixes for the fabrication of MMMs [11]. The versatility of their fabrication methods, along with their low ecological footprint and cost, make cellulose-based membranes difficult to surpass in various applications. Cellulose acetate (CA) has been repeatedly applied to prepare ultrafiltration and nanofiltration membranes because of its environmental friendliness, low price, moderate chlorine resistance, good biocompatibility, and high hydrophilicity [12,13]. However, CA membranes exhibit poor mechanical strength, low chemical resistance, thermal stability, sensitivity to cleaning agents, and vulnerability to fouling resistance; hence, the desired separation/purification yields cannot be achieved. These limitations can be overcome by incorporating additives into the membranes’ structures, either in bulk or on the surface, through a composite approach (i.e., mixed matrix membranes (MMMs)). CA-based MMMs extend the applications of CA and are suitable for different separation processes, including gas separation.

Numerous fillers have been incorporated into CA to fabricate MMMs for different applications. For example, 20 wt.% of micro-sized nano-porous sodium zeolite-Y (NaY) particles were incorporated into a CA membrane for CO_2_/N_2_ separation, resulting in a twofold increase in CO_2_ permeability and no considerable decrease in CO_2_/N_2_ selectivity [14]. Similarly, when 0.1 wt.% of functionalized multiwalled carbon nanotubes (MWCNTs-F) were loaded onto CA using the wet-phase inversion technique for CO_2_/N_2_ separation, excellent permeance and selectivity, with a homogenous dispersion of MWCNTs-F into the CA matrix, were achieved [15]. NH_2_-MIL-53 (Al) filler has also been immersed into CA polymer for CO_2_/N_2_ and CO_2_/CH_4_ separation. The fabricated MMMs showed a 200% improvement in CO_2_ separation performance compared to pristine CA [16]. MMMs have been developed based on CA, graphene oxide (GO) nanosheets, and Fe_3_O_4_ magnetic nanoparticles for anionic dye removal. The 0.2 wt.% nanocomposites exhibited 100% rejection for all of the tested anionic dyes (Acid Blue 7, Reactive Red 120, and Direct Red 23) at pH 9 [17]. In another study, CA-based MMMs were synthesized using a mixed metal oxide nanoparticle–polymer composite (Fe–Al–Mn@chitosan) with the nanofiller dispersed in the CA matrix to remove fluoride from groundwater. Excellent defluoridation performance was obtained along with an antibacterial effect [18]. CA-based MMMs have been fabricated with zwitterionic polydopamine-sulfobetaine methacrylate or P(DA-SBMA) nanoparticles through wet-phase inversion for the treatment of oily wastewater and showed 95–99% oil separation efficiency [19]. In another study, α-aminophosphonate modified montmorillonite (MMT) and Ag-TiO_2_ nanoparticles have been used with CA polymer to fabricate MMMs for treating textile wastewater. The hydrophilicity, mechanical stability, and performance of the fabricated membranes were enhanced and they demonstrated good rejection of hazardous contaminants (chromium, nickel, lead, and fluoride) [20]. In other work, linde type A (LTA) zeolite nanoparticles (ZNPs) were immersed in CA using a dry–wet-phase inversion technique for reverse osmosis desalination. The resultant MMMs with 0.07 g ZNPs showed superior salt rejection (95.5%) and high permeate water flux (1.3 L/m^2^·h) compared to a pristine CA membrane [21,22].

Recently, magnetic Fe-Ni alloy nanostructures were prepared with different particle sizes and exhibited attractive structures and superior ferromagnetic properties [23]. This makes them a promising kind of inorganic filler that can be incorporated into CA matrixes to produce magnetic MMMs with attractive characteristics that can be used in various applications. These magnetic fillers have been successfully incorporated in poly(ethersulfone) (PES) membranes [24]. The same fillers were used in this study but a different polymeric matrix—cellulose acetate (CA)—was used. The effects of the various characteristics of the different polymeric matrixes on the structure, properties, and performance of the different magnetic mixed matrices are discussed and highlighted.

In this study, magnetic CA-based MMMs were synthesized through incorporation of magnetic Fe-Ni alloy nanostructures into CA polymer blended with poly(methylmethacrylate) (PMMA) and lithium chloride (LiCl) in order to improve the mechanical properties and porosity of the fabricated membranes. The blank CA and magnetic CA-based MMMs prepared were characterized using different analysis techniques, such as scanning electron microscope (SEM) imaging, scanning-transmission electron microscope (STEM) imaging, energy-dispersive X-ray (EDX) analysis, thermogravimetric analysis (TGA), X-ray diffraction (XRD) analysis, and Fourier-transform infrared (FTIR) analysis. Furthermore, the static water contact angle, membrane thickness, surface roughness, and membrane porosity (using ethanol and water) were examined; the membrane tensile strength and oxygen transition rate (OTR) were studied; and vibrating sample magnetometer (VSM) analysis was conducted.

## 2. Materials and Methods

### 2.1. Materials

The polymers used for membrane preparation included cellulose acetate (average Mn ~50,000 by GPC), obtained from Aldrich (Munich, Germany), and poly(methylemethacrylate), purchased from Lanxess (Liyang, China). The solvent mixture used included 1,4-dioxan (99.5%), purchased from Adwic, El-Nasr Pharmaceutical Chemicals Company (Qalyubia, Egypt); acetone (99%), obtained from El-salam for Chemical Industries (Giza, Egypt); methanol (99.8%, HPLC grade), obtained from Fisher (Loughborough, UK); and acetic acid (99.8% for biochemistry), purchased from Acros Organics (New Jersey, USA). Diethyle phthalate (99%) was obtained from Loba Chemie (Mumbai, India). Lithium chloride anhydrous was purchased from Fisher (Loughborough, UK). The chemicals used for alloy synthesis included nickel chloride hexahydrate (NiCl_2_·6H_2_O, 98%) and ferrous chloride tetrahydrate (FeCl_2_·4H_2_O, 99.99%) as sources of metal ion, which were purchased from Sigma (Darmstadt, Germany). Hydrazine hydrate (N_2_H_4_·H_2_O, 99%) was obtained from Fisher (Horsham, UK) as a reducing agent. Sodium hydroxide (NaOH, 98%) catalyst was purchased from Trading Dynamic Co. (TDC, Cairo, Egypt). Distilled water was used as a solvent.

### 2.2. Methods

#### 2.2.1. Preparation of the Iron–Nickel Magnetic Alloys/Fillers

Nanocrystalline Fe_10_Ni_90_ and Fe_20_Ni_80_ alloys were synthesized as described in a previous study [23]. Briefly, 80 mL of aqueous solutions of Fe^2+^ and Ni^2+^ ions with molar ratios 1:9 and 2:8 were prepared by dissolving appropriate amounts of FeCl_2_·4H_2_O and NiCl_2_·6H_2_O in distilled water. The prepared solution was vigorously stirred in a magnetic stirrer equipped with a heating unit at 1400–1600 rpm and 95–98 °C. Then, the metal solution was reduced by the second solution of warm aqueous hydrazine (N_2_H_4_·H_2_O, 99 wt.%) and aqueous NaOH (0.1 M) at pH 12.8 and a volumetric ratio of about 5:1. The precipitation of fine black alloys was separated magnetically, and then they were washed repeatedly with distilled water until neutral pH was obtained and dried in a vacuum oven at 35 °C for 24 h.

#### 2.2.2. Preparation of Cellulose Acetate (CA) Polymeric Dope

Cellulose acetate (CA) dope was prepared by mixing equal volumes of solution one (wt.%: 18 CA in 44.3 dioxane, 16.4 acetone, 8.2 acetic acid, and 13.1 methanol solvents) and solution two (wt.%: 18.6 CA and 1.6 PMMA in 37.3 dioxane, 18.6 acetone, 18.6 dimethyleformamide, and 5.3 diethylphthalate). Then, 1 wt.% lithium chloride was added to the mixed solutions and they were stirred at 40 °C for 24 h, after which a viscous homogeneous solution was formed.

#### 2.2.3. Preparation of Magnetic Mixed Cellulose Acetate Matrix Membranes

The prepared magnetic alloys (2 wt.% of either Fe_10_Ni_90_—starfish-like or Fe_20_Ni_80_—necklace-like) were first dispersed in 5 mL of methanol using ultrasonication for 15 min at room temperature. Then, the alloy solution was added to the polymeric dope with heating at 40 °C and mixed gently (without using a magnet), and the air bubbles were removed from the composite solution by degassing for 5 min. Immediately, the MMMs were cast on a glass plate using a doctor casting knife adjusted to 350 µm thickness and then immersed in 2.5 L of distilled water with 0.1 wt.% methanol at room temperature for 2 h. Then, the prepared MMMs were washed using distilled water with immersion and flushing and dried in atmospheric air at room temperature. Table 1 shows the coded names and the compositions of the prepared magnetic mixed cellulose acetate (CA)-matrix membranes.

#### 2.2.4. Iron–Nickel Alloys and Membrane Characterization

The amount of monolayer adsorption (V_m_), adsorbate (alloys/fillers) surface area (a_s_), total pore volume, and average pore diameter of the prepared iron–nickel Fe_10_Ni_90_—starfish-like and Fe_20_Ni_80_—necklace-like alloys fabricated and used in this work were determined using gas adsorption measurements (Microtrac MBR, Belsorp mini X version, Osaka, Japan). Nitrogen gas was used in the test.

The porosities (ε) of the fabricated blank CA and magnetic mixed CA matrix membranes were estimated using the dry–wet weight method [25]. The wet weight of the membrane sample was measured after immersing it in both distilled water and absolute ethanol at room temperature for 24 h. After that, the samples were dried using an oven at 60 °C for 24 h and the dry weight of the samples was measured. The membrane porosity was determined using the following equation:

$$\varepsilon =\frac{\frac{W_{w}-W_{d}}{\rho_{e}}}{\frac{W_{w}-W_{d}}{\rho_{e}}+\frac{W_{d}}{\rho_{p}}}\times 100$$

where *W_W_* is the wet membrane weight (g), *W_d_* is the dry membrane weight (g), *ρ_e_* is the density of ethanol or distilled water (g/cm^3^), and *ρ_P_* is the density of the polymer or polymer blend (g/cm^3^).

The static water contact angle of the magnetic mixed CA matrix membrane samples was measured using a Gonimeter model 500-F1 coupled with a video camera and image analysis software. A water droplet of 7 μL was dropped on different spots of the membrane surface. The membrane samples were analyzed using the captured images at consecutive time frames, the right and left contact angles were estimated using the image analysis software, and the mean value was determined. The reported value was the average of eight readings from three different samples for each mixed CA matrix membrane.

The average membrane thickness was calculated as an average of eight measurements at different points on three different membrane samples using a digital micrometer (Dsqua, 0–25 mm/0.001 mm, Cornegliano Laudense, Italy).

The average roughness of the prepared mixed CA matrix membranes was estimated using a surface roughness tester (SJ-201 P, Mitutoyo, Kawasaki, Kanagawa, Japan). The membrane surfaces were fixed onto a glass plate before measuring. The calibration of the instrument was undertaken by measuring the roughness of the glass plate used. The recorded results are the average of eight measurements from four different membrane samples.

X-ray diffraction (XRD) was employed to characterize the synthesized alloys and the magnetic mixed CA matrix membranes. XRD measurements were carried out on a Shimadzu XRD-7000 diffractometer (Kyoto, Japan, 45 kV, 30 mA; CuKα + Ni-filtered radiation, λ = 0.15406 nm). The 2θ range was 5–80°, with a scanning rate of 4°/min and a scanning step of 0.026°.

Thermogravimetric studies of the prepared membranes were carried out using a thermogravimetric analyzer (Shimadzu TGA-50, Nishinokyo Kuwabara-cho, Nakagyo-ku, Kyoto, Japan). The samples were scanned over a temperature range from room temperature (25 °C) to 1000 °C at a temperature gradient of 10 °C/min under 40 mL/min nitrogen flow.

FTIR-ATR analysis of the fabricated blank and magnetic mixed CA matrix membranes was conducted using an FT-IR spectrophotometer (Shimadzu FTIR-8400S, Nishinokyo Kuwabara-cho, Nakagyo-ku, Kyoto, Japan) equipped with an ATR accessory in the range from 400 to 1800 cm^−1^. The samples were used after complete drying in air for 72 h.

The prepared magnetic mixed CA matrix membranes were cut in a dumbbell shape. The length of each membrane was 37 mm, and the gauge length of the membranes was about 16 mm; the width was 13 mm at the top and 7.2 mm (narrowest) at the middle of the membrane, which was intended to force a fracture in the middle of the sample. Tensile testing of the films was performed with a Texture Analyzer T2 (Stable Micro Systems, Ltd., Surrey, UK) at a constant crosshead speed of 0.1 mm/s. Stress–strain curves were calculated from the load–elongation curves measured for five samples from two membranes prepared from two prepared dopes for each membrane composition.

To measure the membrane tensile strength, the synthesized magnetic mixed CA matrix membranes were cut using a very sharp shaving blade and then coated with gold and imaged at a voltage of 20 kV and a resolution of 1280 × 960 pixels using scanning electron microscopy (Joel Jsm 6360LA, Akishima, Japan). For cross-section imaging, the membrane samples were immersed in liquid nitrogen and were fractured in order to process them for gold coating before imaging. The chemical compositions were estimated with an area analysis using an energy-dispersive X-ray spectroscopy (EDX) system equipped with SEM.

The fabricated magnetic mixed CA matrix membranes were inspected using a transmission electron microscope (TEM, 2100Plus, JEOL Ltd., Tokyo, Japan) operated at 200 kV. Membrane samples were frozen inside epoxy blocks (Epon 812 Embedding Resin (Mollenhauer, Germany)), and a very thin layer was cut using PowerTom Ultramicrotomes (RMS Boeckeler, Boeckeler Instruments Inc., Tucson, AZ, USA). The chemical compositions were estimated with an area analysis using an energy-dispersive X-ray spectroscopy (EDX) system equipped with STEM.

A vibrating sample magnetometer (VSM, Lake Shore 7410, Woburn, Massachusetts, USA) was used to measure the room-temperature magnetic properties of the nanostructured iron–nickel alloys and the fabricated blank and magnetic mixed CA matrix membranes. The applied field was −20 ≤ H ≤ 20 kOe. For magnetization measurements, the membranes were tied and fixed in a small cylindrical plastic holder between the magnetic pools.

The oxygen transmission rate (OTR) was measured using an N530-B gas permeability analyzer from GBPI Equipment Co., Ltd. (Guangzhou, China) according to ISO standard 15105-1. The ASTM D1434, YBB00082003, JISK7126-A, and GB/T 1038 standards were used for the evaluation of the oxygen gas transmission rate (OTR) with the differential pressure method. Briefly, the membrane was fixed in the middle of the test chamber to separate the chamber into an upper room and a lower room while keeping a constant pressure difference; the initial pressures for the upper and lower rooms were 100 Kpa and 10 Pa, respectively. Oxygen gas molecules could penetrate through the sample from the higher pressure room into the lower pressure room. Gas permeability was measured by detecting the pressure change in the lower pressure room and calculating the gas transmission rate.

## 3. Results

### 3.1. Iron–Nickel Alloys/Fillers

**Figure 1:**
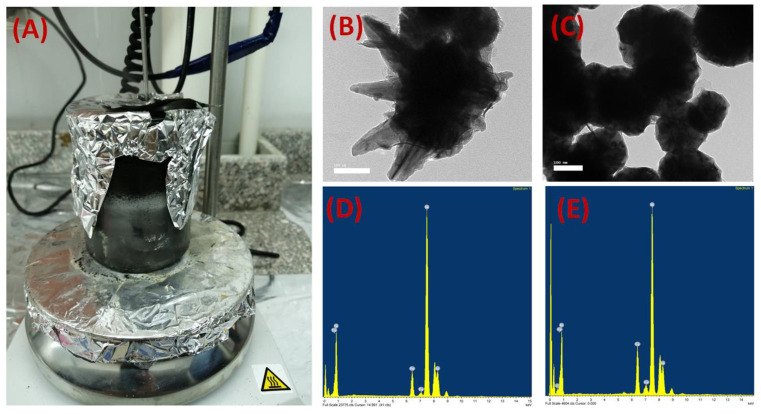
Photos of the prepared iron–nickel alloys during the reduction process (**A**), TEM images of the prepared Fe_10_Ni_90_—starfish-like alloy (**B**) and Fe_20_Ni_80_—necklace-like alloy (**C**), and EDX analysis of the prepared alloys ((**D**) and (**E**), respectively).

Oxygen-enriched air can lower the required capital and the operating costs, reduce the carbon dioxide emissions, and increase the process efficiency. In the present study, flat-sheet mixed cellulose-based matrix membranes were prepared using a solution-casting and phase-inversion method. The composition and purity of the Fe_10_Ni_90_—starfish-like and Fe_20_Ni_80_—necklace-like alloys used were analyzed with X-ray diffraction and according to data published in previous work [23]. Figure 1 shows photos of the prepared iron–nickel alloys during the reduction process (A), TEM images of the prepared Fe_10_Ni_90_—starfish-like alloy (B) and Fe_20_Ni_80_—necklace-like alloy (C), and the EDX analysis of the prepared alloys (D and E, respectively) using TEM equipment.

The TEM imaging and EDX analysis confirmed the formed shape and composition of the prepared alloys.

Table 2 shows the characteristics of the type III isotherm obtained for the two prepared iron–nickel alloys, showing the typical features of non-porous materials. The obtained results confirmed the presence of minimum adsorption between the prepared alloys and nitrogen gas that was used for the Brunauer–Emmett–Teller (BET) analysis.

### 3.2. Magnetic Mixed Cellulose Acetate (CA) Matrix Membranes

**Figure 2:**
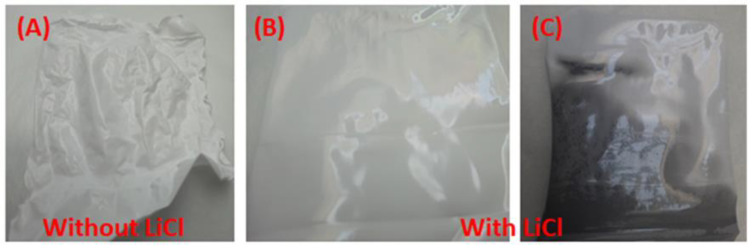
Photos illustrating the effect of the addition of lithium chloride (LiCl) on shrinkage in the prepared blank cellulose acetate (CA) and magnetic mixed CA matrix membranes after drying in atmospheric air. The blank CA without (**A**) and with (**B**) LiCl and the magnetic mixed CA matrix membrane with LiCl (0.1 wt.%) (**C**).

The addition of lithium chloride (LiCl) to the membrane dope increased its viscosity due to LiC1–solvent interaction [26]. This interaction caused an increase in the rate of polymer precipitation during the immersion step. On the other hand, the presence of LiC1 entrapped inside the polymer chains resulted in the retention of the absorption of moisture from the air; the amount of absorbed water was meager and could not alter the bulk properties of the dope, which minimized the shrinking of the polymer chain during the drying process, as shown in Figure 2A (blank cellulose acetate (CA) membrane without use of LiC1 additive). The addition of LiCl affected the formation of the flat membranes, as shown in both the blank and mixed CA matrix membranes (Figure 2B and Figure 2C, respectively). Figure 2C shows the change in the magnetic mixed CA matrix membrane during the drying step in atmospheric air. 

**Figure 3:**
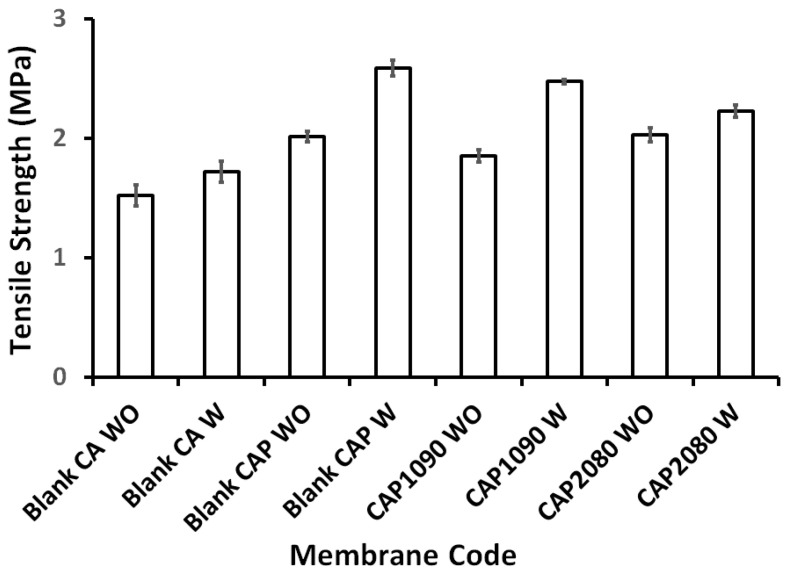
The effects of the added poly(methylmethacrylate) (PMMA) on the tensile strength (MPa) of the prepared blank cellulose acetate (CA) and magnetic mixed CA matrix membranes in the absence and presence of 0.1 wt.% of lithium chloride in the membrane dope.

As shown in Figure 3, the addition of poly(methylmethacrylate) (PMMA) to the membrane dope resulted in improvements to the tensile strength of all the prepared membranes in the range of 21 to 50%. Moreover, the added PMMA clearly improved the membranes’ surface properties: they became less stiff and, consequently, their handling was improved. For this improvement, the PMMA was used in the dope composition. On the other hand, the synergy between the added PMMA and the lithium chloride had a positive effect on the mechanical strength of the fabricated blank CA and magnetic mixed CA matrix membranes. 

### 3.3. Membrane Porosity and Thickness

**Figure 4:**
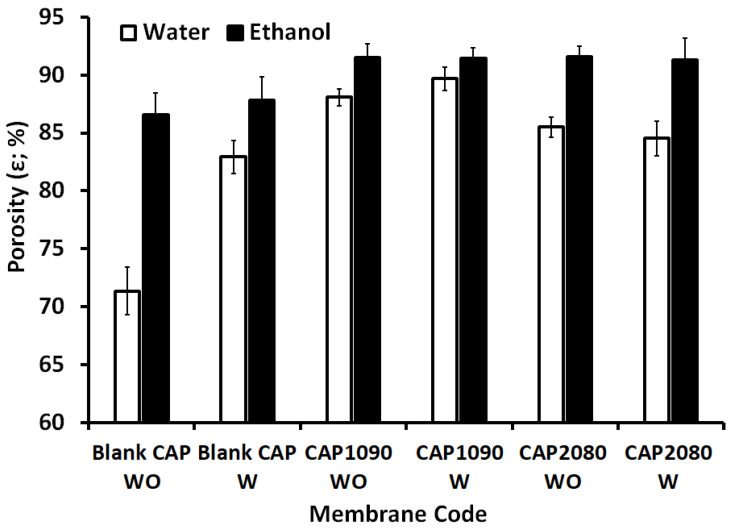
Porosity (%) of the blank cellulose acetate (CA) and the magnetic mixed CA matrix membranes with the absence and presence of 0.1 wt.% of lithium chloride in the membrane dope measured using absolute ethanol (black bars) and water (white bars). For all the prepared membranes, 0.8 wt.% poly(methylmethacrylate) (PMMA) was used.

According to our previous work [24], measurements of porosity using distilled water can be achieved with large-size pores, whereas measurements of porosity using ethanol can be achieved with all ranges of pores. As shown in Figure 4, the effect of lithium chloride on the large pores was pronounced, especially in the blank CA membrane, in which an increase of around 16% was observed in the porosity measured with water. This effect was amplified with the addition of the iron–nickel alloys (fillers), with up to 23% and 20% increases with the embedded Fe_10_Ni_90_—starfish-like and Fe_20_Ni_80_—necklace-like alloys/fillers, respectively. All the magnetic mixed CA matrix membranes showed around 91% total porosity.

Compared to a PES matrix [24], the total porosity of the magnetic mixed CA matrix membranes was in the same range (91–93%). The large-size pores in the magnetic mixed CA matrix membranes seemed to be about 10% and 17% larger than in the magnetic mixed PES matrix membranes with the absence and presence of lithium chloride in the membrane dopes. This can be explained by the CA matrix (the contact angle of the blank CA was around 50°) being more hydrophilic than the blank PES (the contact angle of the blank PES was around 80°) matrix, which facilitated the entrance of the water into the demixing process and, consequently, increased the creation of large-size pores and resulted in much easier removal of the lithium chloride leaving through the large-size pores.

The doctor knife was adjusted to 350 µm as-casting thickness. After coagulation of the membrane dope, around 55% of the as-cast thickness was lost in all the fabricated membranes; all the membranes were around 160 ± 2 µm. No significant differences in the measured membrane thickness as a function of the different alloys’ morphology and/or the concentrations of the used condition were found in this work.

### 3.4. Membrane Microstructure

**Figure 5:**
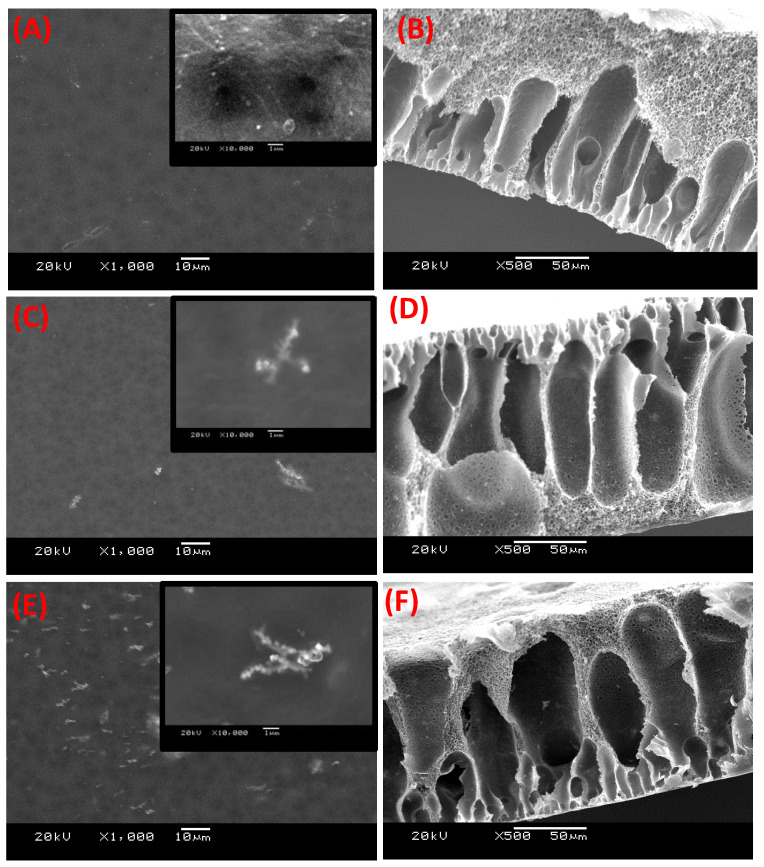
SEM images of the blank cellulose acetate (CA) (**A**) and magnetic mixed CA matrix membranes prepared using Fe_10_Ni_90_ and Fe_20_Ni_80_ alloys, respectively (**C**,**E**). Further, 0.1 wt.% of lithium chloride and 0.8 wt.% of poly(methyl methacrylate) (PMMA) were used. The images were taken at ×1000 and ×10000 magnifications. The SEM cross-sections of the blank CA (**B**) and magnetic mixed- CA matrix membranes prepared using Fe_10_Ni_90_ and Fe_20_Ni_80_ alloys, respectively (**D**,**F**), were imaged at ×500 magnification.

The electron microscope images of the membranes’ surfaces for both the blank CA and the mixed CA matrix membranes are shown in Figure 5. The blank CA membrane appeared with a dense top layer without alloys (fillers). The images of the magnetic mixed CA matrix surfaces reveal that the magnetic alloys were dispersed in the CA matrix. The embedded alloys appeared bright for both the Fe_10_Ni_90_ and Fe_20_Ni_80_ magnetic alloys (Figure 5C,E). This brightness of the alloys was enhanced with the projection of a tiny part on the membrane surface. There was no porosity on the membrane surface but there was a bit of porosity at the back of the membrane, which was in full agreement with the previous work [24] on PES matrix. Another observation was that the amount of the magnetic Fe_20_Ni_80_ alloy on the membrane surface (Figure 5E) was larger than that for the magnetic Fe_10_Ni_90_ alloy (Figure 5C).

The cross-section images shown in Figure 5 illustrate the formation of an asymmetric structure composed of a skin layer over a more open porous structure (i.e., selective and supporting layers). The change in the color of the top and the bottom of the magnetic mixed CA matrix membranes suggested that much of the alloys had segregated into the skin layer. Although some fillers were noticed through the membrane cross-section, they seemed to be completely coated by the used CA matrix. 

The cross-section illustrated the long porous area next to the dense skin layer and a tiny pore network structure around the larger pores [26,27]. This structure was noticeable in all the fabricated magnetic mixed CA matrix membranes. The formation of finger-like cavities in the membranes was associated with a high rate of precipitation of the polymer from the casting solution upon immersion in the coagulation bath (non-solvent), which was caused by the strong tendency of the used solvent to mix with the non-solvent. The addition of lithium chloride to the membrane dopes causes the formation of larger cavities [28]. Lithium chloride generated a denser surface structure due to the formation of complexes with the used solvent mixture, which significantly increased the dope solution’s viscosity, leading to the surface layer becoming denser [29].

**Figure 6:**
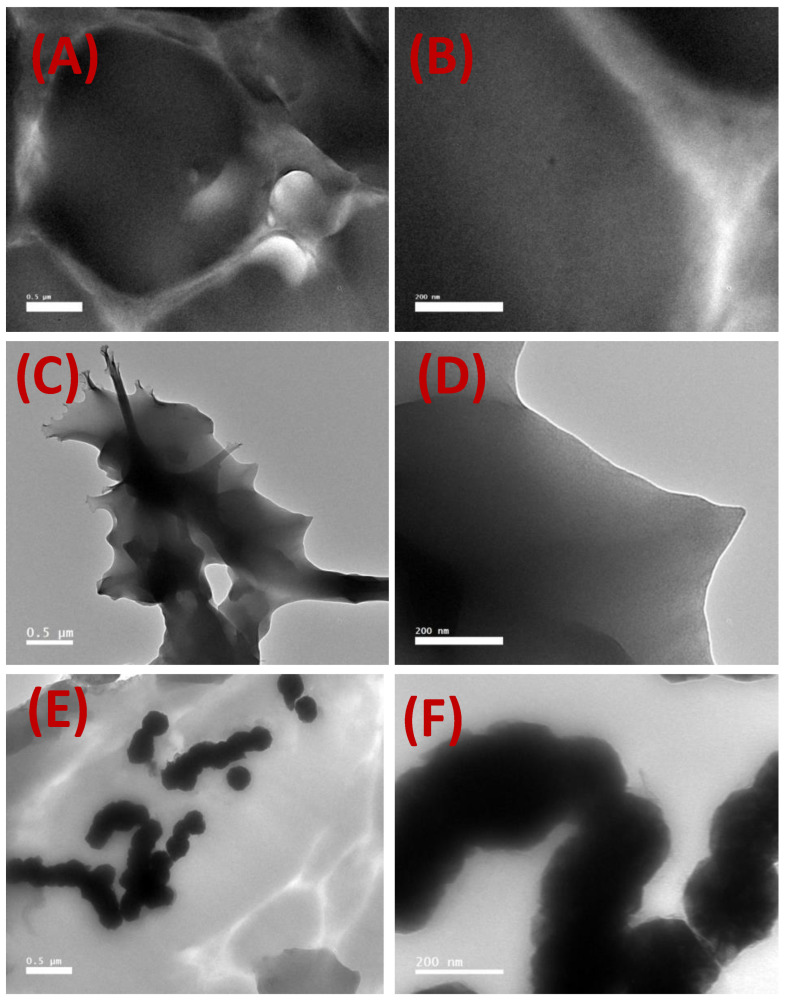
TEM images (0.5 µm and 200 nm bar scale) of the blank cellulose acetate (CA) (**A**,**B**) and the magnetic mixed CA matrix membranes prepared using Fe_10_Ni_90_ (**C**,**D**) and Fe_20_Ni_80_ alloys (**E**,**F**), respectively, inside the epoxy blocks.

TEM images of the blank CA and the magnetic mixed CA matrix membranes are shown in Figure 6. The blank CA membrane did not contain any fillers. Figure 6C–F confirm the retention of the same morphologies for the Fe_10_Ni_90_—starfish-like and Fe_20_Ni_80_—necklace-like iron–nickel alloys embedded inside the magnetic mixed CA matrix membranes. However, it seems the alloys were embedded more deeply inside the CA polymeric matrix compared to the previous work using a PES polymeric matrix [24]. Moreover, there was no alignment in the filler directions as observed in the PES polymeric matrix [24], and the filler distribution of the embedded alloys/filler was homogenous all over the magnetic CA mixed-matrix membranes.

### 3.5. The Elemental Composition

It can be observed from Table 3 that the blank cellulose acetate (CA) membrane contained only oxygen and carbon when lithium chloride was not used in the membrane dope and other traces of chloride when lithium chloride was used in the membrane dope (the detector of the EDX used did not detect lithium). The magnetic mixed CA matrix membranes contained oxygen, carbon, iron, nickel, and a trace of chloride. The low iron and nickel detection may be attributable to the embedding of the alloy inside the membrane (i.e., through the membrane cross-section), as was observed in the imaging. The EDX equipment equipped with TEM (Table 4) gave a more clear indication of the inclusion of the iron–nickel alloys inside the magnetic CA mixed-matrix membranes, although with much lower beam thickness.

### 3.6. Membrane Hydrophilicity

**Figure 7:**
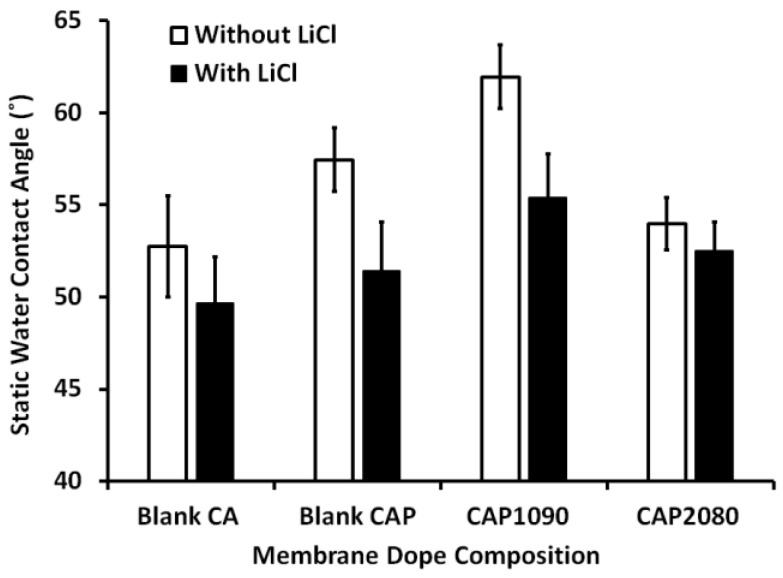
Static water contact angle in the composition of the blank CA and the different magnetic mixed CA matrix membranes in the absence (black bars) and presence (white bars) of 0.1 wt.% of lithium chloride in the membrane dope.

The static water contact angles for both the blank CA and magnetic mixed CA matrix membranes were measured and are presented in Figure 7. The addition of 0.1 wt.% lithium chloride into the blank CA membrane resulted in about a 10% reduction in the measured static water contact angle. In the absence of lithium chloride, the addition of a low percentage (0.8 wt.%) of PMMA resulted in a slight increase (about 7%) in the measured static water contact angle, which can be attributed to the relative hydrophobicity of the PMMA relative to the CA. The addition of magnetic iron–nickel alloy resulted in a change in the measured static water contact angle as a function of the iron–nickel alloy morphology used, with incorporation of Fe_10_Ni_90_ alloy reflected by an increase in the static water contact angle (about 15% and 7% increases compared to the blank CA and the blank CAP, respectively), whereas incorporation of Fe_20_Ni_80_ caused a decrease in the measured static water contact angle by around 7% compared to the blank CA membrane. In other words, adding the Fe_20_Ni_80_—neck-lace-like alloy canceled the effect of adding the PMMA on the measured static water contact angle. This can be attributed to the effect of surface roughness, with the contact angles for droplets placed on a rough surface wetting only a portion of the surface and most probably being larger than the contact angles on the smooth surface, which wet the surface more completely. These were related to changes in the fractional contact areas of the water droplets due to the formation of air pockets between the rough surface and the droplet [27]. As in the following measurements, the fillers/alloys added to the CA matrix resulted in an increase in the membrane surface roughness and, consequently, the measured contact angle. The increases in the surface roughness of the magnetic mixed CA matrix were lower in the case of incorporation of Fe_20_Ni_80_ than in the case of incorporation of Fe_10_Ni_90_ alloy, which was reflected in a decrease in the measured static water contact angle. The effect of adding iron–nickel alloys inside the CA matrix on the measured static water contact was minimized when using lithium chloride in the membrane dope, although the same effect was retained with the incorporation of the different alloys/fillers. 

### 3.7. X-ray Diffraction (XRD)

**Figure 8:**
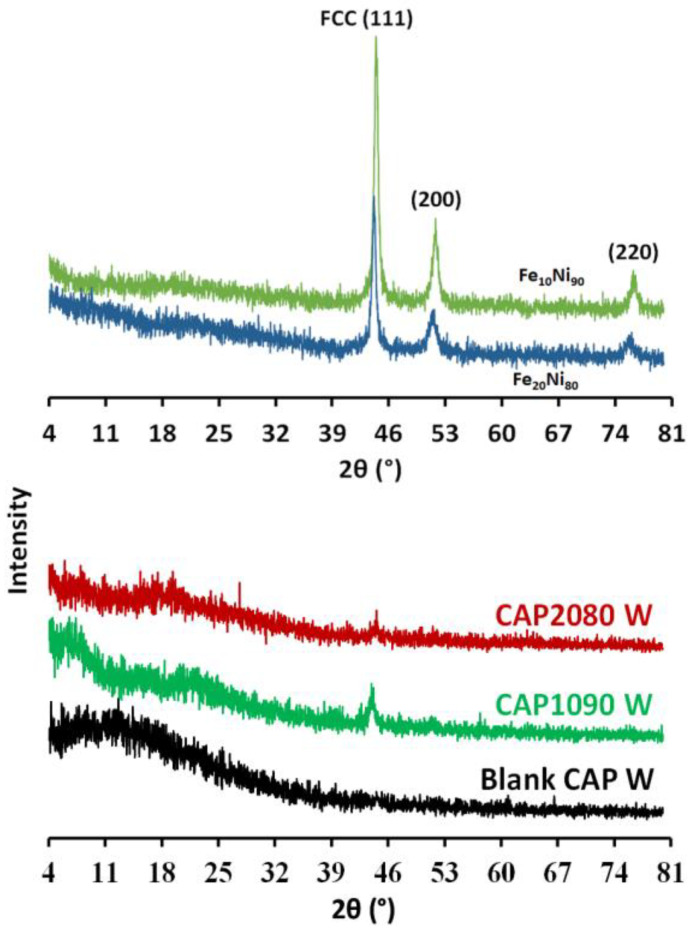
XRD analysis of the magnetic iron–nickel alloys used as fillers, the blank cellulose acetate (CA), and the magnetic mixed CA matrix membranes with 0.1 wt.% of lithium chloride used in the membrane dope.

In Figure 8, the XRD pattern for the cellulose acetate (CA) exhibits a typical broad peak around 2θ = 17° and 21°, indicating semicrystalline CA. The peaks located at 44.41°, 51.71°, and 76.21° can be indexed to the (111), (200), and (220) planes of the crystalline face-centered cubic (fcc) FeNi_3_ alloys [23]. The XRD pattern for the magnetic mixed CA matrix membranes showed the presence of the broad peak for the CA, as well as peaks located at 44.41° that could be indexed to FeNi_3_ alloys, indicating the successful impeding of the iron–nickel alloys inside the CA matrix. The obtained results indicate the successful formation of magnetic mixed CA matrix membranes.

### 3.8. Membrane Roughness

**Figure 9:**
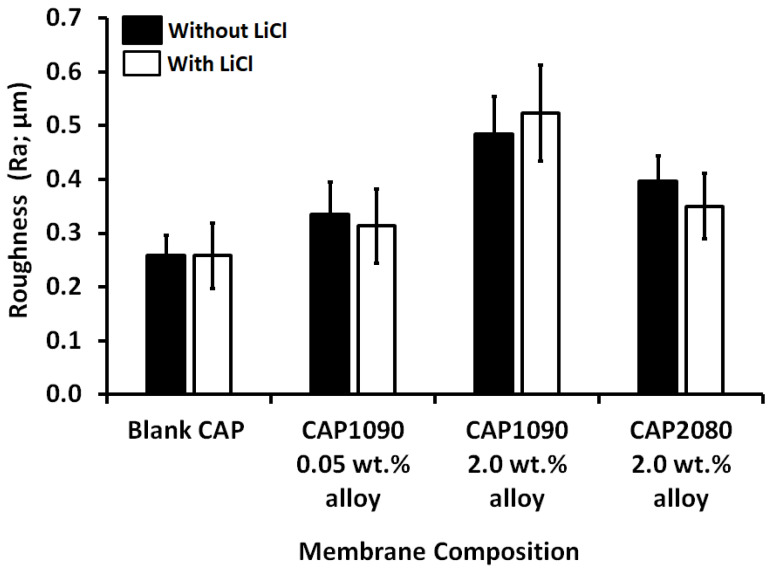
Membrane roughness (Ra) of both the blank CA and the magnetic mixed cellulose acetate (CA) matrix membranes as a function of membrane dope composition (wt.% alloy concentration) with the absence (black bars) and presence of 0.1 wt.% of lithium chloride (white bars). Poly(methyl methacrylate) (PMMA; 0.8 wt.%) was used in the membrane dope for all the prepared blank CA and magnetic mixed CA matrix membranes.

The arithmetic average of the roughness profile (Ra) was calculated as the average roughness of the surfaces measured from microscopic peaks and valleys. As shown in Figure 9, adding iron–nickel alloys affected the measured membrane roughness, which increased gradually as a function of the Fe_10_Ni_90_ alloy concentration (19% and 100% increases corresponding to 0.05 and 2 wt.% alloys, respectively). The effect of adding Fe_20_Ni_80_ alloy was much weaker (about a 35% increase in the membrane roughness with 2 wt.% alloys). This could have been related to the different morphologies of the two alloys used [23]. As described in the previous section, the change in the membrane roughness was reflected in the static water contact angle measured. Lithium chloride did not significantly affect the roughness of the fabricated mixed CA matrix membranes.

### 3.9. Fourier-Transform Infrared Spectroscopy-Attenuated Total Reflectance (FTIR-ATR) Analysis

**Figure 10:**
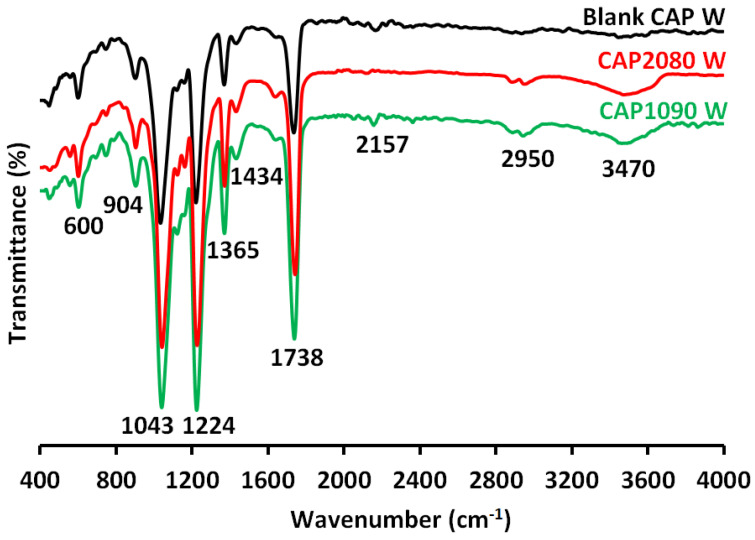
Fourier-transform infrared spectroscopy-attenuated total reflectance (FTIR-ATR) analyses of the blank cellulose acetate (CA) membrane (Blank CPA W) and the magnetic mixed CA matrix; CAP1090 W and CAP2080 W membranes. For the membrane dope, 0.1 wt.% of lithium chloride and 0.8 wt.% poly(methylmethacrylate) (PMMA) were used.

FTIR-ATR spectra of the blank cellulose acetate (CA) and magnetic mixed CA matrix membranes are shown in Figure 10. The widening of absorption peaks was generally observed in the region from 3300 to 3700 cm^−1^, corresponding to the hydrogen bonds of hydroxyl groups (-OH) resulting from the presence of moisture in the sample. The absorption peak at the wavenumber of 2950 cm^−1^ corresponded to the stretching of -CH- of methyl groups (-CH_3_). The carbonyl group (C=O) stretching vibration was at 1738 cm^−1^. The -CH_2_- deformation vibration occurred at 1434 cm^−1^. The characteristic peaks of the C-O-C anti-symmetric stretching vibrations of the ester group of CA were at 1224 cm^−1^. The -C-OH stretching vibration of pure CA occurred at the wavenumber of 1039 cm^−1^. The presence of the absorption peaks at 904 cm^−1^ in the pure CA could have been due to the combination of -C-O stretching and -CH_2_- rocking vibrations.

### 3.10. Thermogravimetric Analysis (TGA)

**Figure 11:**
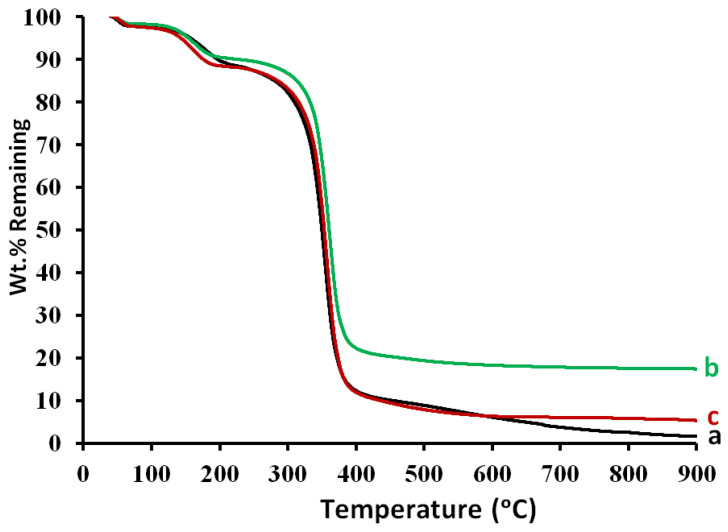
TGA of the blank cellulose acetate (CA) membrane ((a) blank CAP W) and the magnetic mixed CA matrix membranes ((b) CAP1090 W and (c) CAP2080 W). For the membrane dope, 0.1 wt.% of lithium chloride and poly(methylmethacrylate) (PMMA; 0.8 wt.%) were used.

The weight loss in the temperature around 40–120 °C shown in Figure 11 can be attributed to the loss of the residual volatile solvent and elimination of the adsorbed moisture. The weight loss in the temperature range of 300 to 400 °C can be attributed to the degradation of the CA chains. The weight loss in the temperature around 300–900 °C can be attributed to the complete degradation of the alloys, leaving residue ash traces in the blank CA membrane (line a; 1.6% remaining at 900 °C). The mixed CA matrix membranes seemed to contain iron–nickel alloys that could resist the effects of temperature, maintaining 17.4 and 5.3 wt.% and remaining at 900 °C for the embedded Fe_10_Ni_90_ and Fe_20_Ni_80_ alloys, respectively. This confirmed the positive thermal stability of the magnetic mixed CA matrix membranes.

The final remaining wt.% in the TGA curve was different for the two iron–nickel alloys embedded in the CA membranes, which can be explained by the fact that the CAP1090 W membrane was more thermally stable than the CAP2080 W.

### 3.11. Vibrating Sample Magnetometer (VSM) Analysis

**Figure 12:**
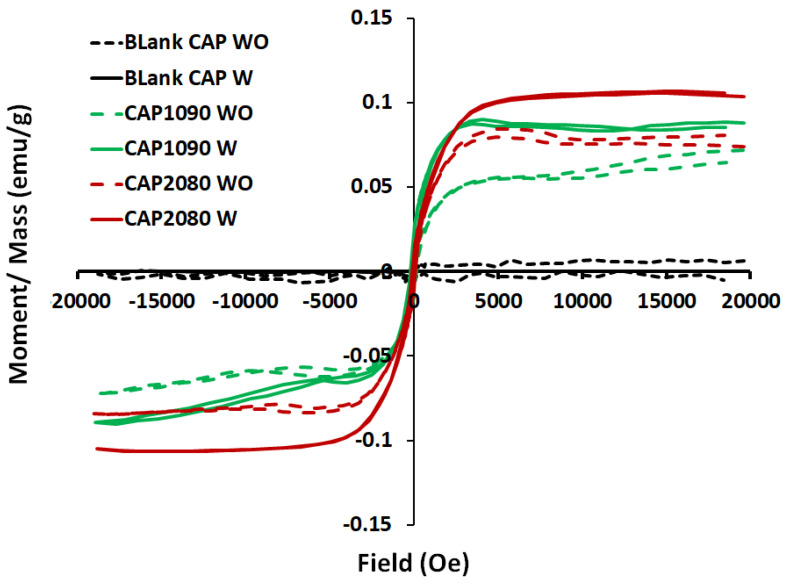
The M-H hysteresis loops of the blank cellulose acetate (CA) and the magnetic mixed CA matrix membranes without and with the addition of 0.1 wt.% of lithium chloride in the membrane dopes. Poly(methylmethacrylate) (0.8 wt.%) was used in the membrane dope of all the prepared membranes.

The magnetic mixed cellulose acetate (CA) matrix membranes took the form of an S-shape, but the blank CA membranes did not form a clear S-shape, as shown in Figure 12. The results for the magnetic vibration of the fabricated magnetic mixed CA matrix membranes showed higher coercivity (Hc; Oe) than that for the blank CA membranes (about sixfold improvement for the membranes containing Fe_10_Ni_90_ and Fe_20_Ni_90_ magnetic alloys/fillers without the addition of lithium chloride additive and about fourfold and twofold for the membranes containing Fe_10_Ni_90_ and Fe_20_Ni_80_ magnetic alloys/fillers, respectively, with the use of lithium chloride in the membrane dope. 

**Figure 13:**
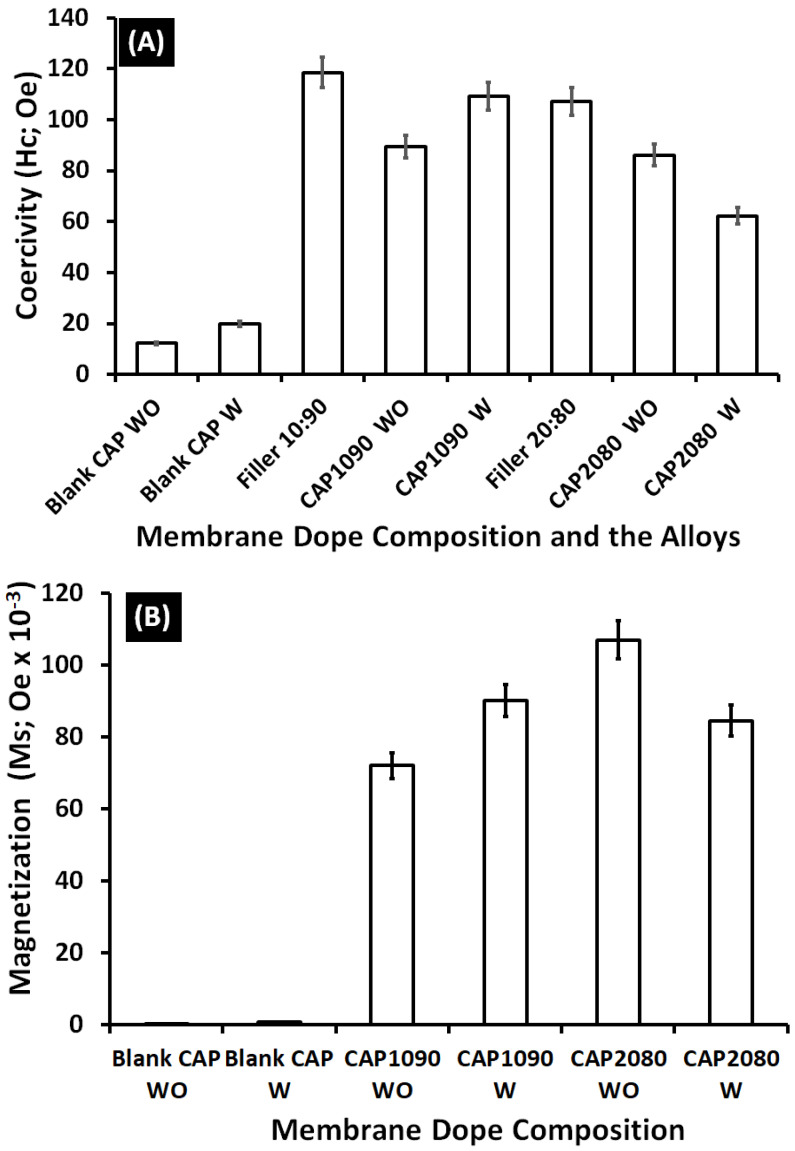
(**A**) Coercivity (Hc; Oe) and (**B**) magnetization (Ms) of the iron–nickel alloys/fillers (Fe_10_Ni_90_ and Fe_20_Ni_80_) as well as the blank cellulose acetate (CA) and the magnetic mixed CA matrix membranes without and with the addition of 0.1 wt.% of lithium chloride in the membrane dope. Poly(methylmethacrylate) (0.8 wt.%) was used in the membrane dope of all the prepared membranes.

On the other hand, the blank CA membranes with and without the addition of lithium chloride Figure 13 showed almost no magnetization properties. For the different iron–nickel alloys, the magnetic mixed CA matrix membranes that contained Fe_20_Ni_80_ showed higher magnetization and lower coercivity than the magnetic mixed CA matrix membranes containing Fe_10_Ni_90_ alloy. High coercivity is required for the targeted application (oxygen-enrichment gas separation without application of an external magnetic field for the separation cell), which highlights the Fe_10_Ni_90_ alloy as having better performance in the fabricated magnetic mixed CA matrix membranes.

### 3.12. Oxygen Transmission Rate (OTR)

Measurement of the amount of oxygen gas that passed through the barrier over a given period was undertaken for the fabricated blank cellulose acetate (CA) and magnetic mixed CA matrix membranes. The blank CA membrane without and with the use of lithium chloride in the membrane dope did not give results for oxygen permeation (0.01 × 10^−5^ cm^3^/(m^2^·s)). The magnetic CA mixed-matrix membrane containing the starfish-like Fe_10_Ni_90_ alloy showed an OTR of 1.271 × 10^−5^ cm^3^/(m^2^·s), about two times the OTR of the magnetic mixed CA matrix membrane containing the necklace-like Fi_20_Ni_80_ alloy. It seems that the Fe_10_Ni_90_ alloy was more efficient in the transmission of oxygen. Compared to the PES polymeric matrix [24], the OTR of the CA polymeric matrix membranes had a lower OTR, which can be attributed to the effect of the tight contact between the hydrophilic CA polymeric matrix and the iron–nickel alloys, which shielded the fillers and negatively affected its affinity with the oxygen. 

## 4. Discussion

Poly(methyl methacrylate) (PMMA) is an optically transparent, hard, thermoplastic polymer, well-known for its high impact strength, light weight, shattering properties, high scratch resistance, and favorable processing conditions. Moreover, PMMA has a high Young’s modulus and a low elongation at break due to the presence of the adjacent methyl group (CH_3_) in the polymer structure, which prevents it from packing closely in a crystalline fashion and from rotating freely around the C-C bonds, hence providing it with weather resistance and scratch resistance. Furthermore, this polymer has reasonable resistance to chemicals, being unaffected by aqueous solutions of most laboratory chemicals [30]. Consequently, we chose to use it in a minor percentage to enhance the mechanical properties of the fabricated magnetic mixed cellulose acetate (CA) matrix membranes.

**Figure 14:**
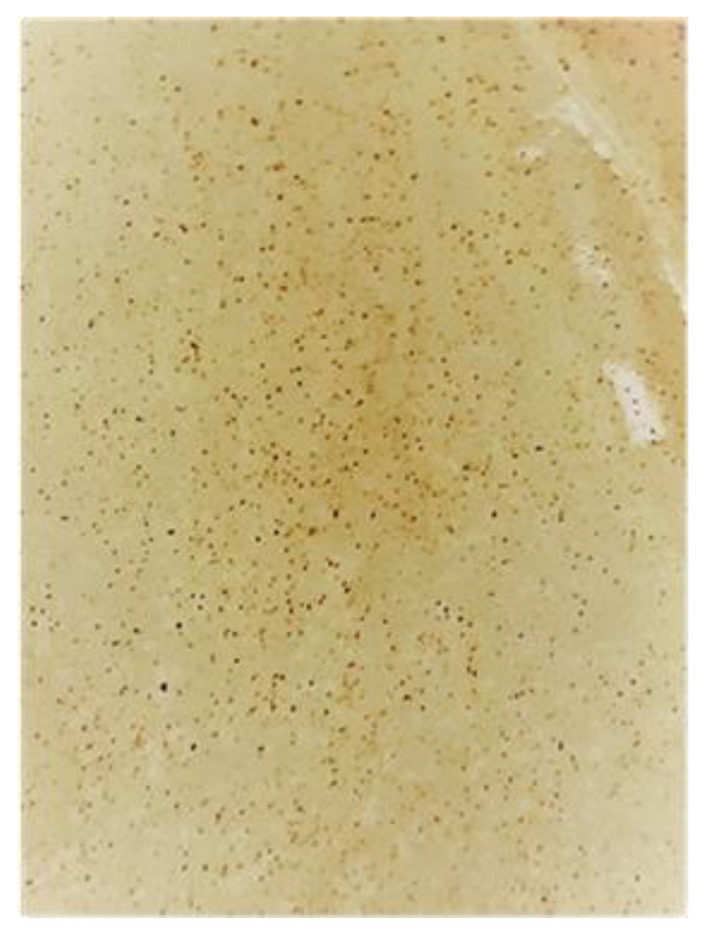
Photo showing the homogeneous distribution of the added Fe_20_Ni_80_ magnetic alloys embedded inside the cellulose acetate (CA) polymer matrix.

The enhanced construct option in ImageJ software showed the good distribution of the Fe_20_Ni_80_—necklace-like alloy/filler embedded inside the CA polymeric matrix (Figure 14).

The low affinity of nitrogen gas in the fabricated iron–nickel alloy was confirmed with a Brunauer–Emmett–Teller (BET) test. The viscosity of the membrane dope seemed to have increased, as noticed during the casting process. This was reflected in the weakening of the pulling of the added magnetic alloy toward the surface, as described in previous work [24] using a poly(ethersulfone) (PES) polymeric matrix. This observation was supported by observations of the filler in the cross-section imaging with SEM (Figure 5). Furthermore, although the embedded alloys appeared as bright for both the Fe_10_Ni_90_ and Fe_20_Ni_80_ magnetic alloys (Figure 5C,E), there was less brightness than in the case of the PES polymeric matrix [24]. This was also noticed in the TEM imaging of the magnetic mixed CA matrix membranes (Figure 6). This can be attributed to the difference in the hydrophilicity of the used polymer; the blank CA (52.7° and 49.7°) was more hydrophilic than the PES (79.5° and 67.6°) [24] with the absence and presence of lithium chloride in the membrane dope, respectively. This facilitated the direct contact (i.e., due to the good compatibility between the surface properties) between the iron–nickel alloys/filler used and the CA polymeric matrix, which inhibited the pulling out of the embedded alloys/filler from the effect of the casting knife. As a result, the alloys/fillers were distributed along the membrane cross-section with the CA polymer, whereas the alloys/fillers were mostly pulled out to the surface with PES polymeric matrix. By using only 0.8 wt.% PMMA in the membrane dope, the static water contact angle of the blank CA increased (57.4° and 51.4°) with the absence and presence of lithium chloride in the membrane dope, respectively. The alloys/filler added into the membrane resulted in a slight increase in the measured static water contact angle (61.9° and 55.4°) in the case of the embedded magnetic Fe_10_Ni_90_ alloy, while the reverse effect was observed in the case of the Fe_20_Ni_80_ magnetic alloy (53.9° and 52.5°), with both the absence and presence of lithium chloride in the membrane dope. This can be attributed to the increase in the surface roughness for the Fe_10_Ni_90_ alloy with its unique cones compared to the Fe_20_Ni_80_ magnetic alloy. The high content of carbon, rather than iron and nickel, in the elemental composition analysis supported the proposal of very good contact between the embedded alloys/filler and the CA polymeric matrix, which led to deep embedding inside the CA matrix. This was supported by the microscope imaging and naked eye observations of the homogenous color through the membrane surface and the lack of orientation of the migrated alloys/filler with the surface, as also shown previously with the PES polymeric matrix [24]. On the other hand, the added PMMA may have mostly solidified on the membrane surface during the demixing process due to its relative hydrophobicity and slightly lower density (1.18 g/cm^3^) compared to the used CA (1.3 g/cm^3^). 

Furthermore, lithium chloride, which was used as a pore former, generated a denser surface structure due to the formation of complexes with the solvent mixture, as well as the effect of PMMA, which can significantly increase the viscosity of a dope solution. This can result in a kinetic hindrance during the phase inversion process, which leads to the surface layer becoming denser and the minimization of the effect of lithium chloride on the surface porosity of the skin layer. On the other hand, the effect of lithium chloride on the dissolution of cellulose and PMMA without chemical changes in the polymeric structure has also been reported [31], which, consequently, affects the solubility of the CA. Moreover, the intermolecular interactions, as well as the presence of the complexation between CA and the residual lithium chloride inside the CA polymeric matrix, may increase a membrane’s ability to attract water, thus promoting membrane wetting and, consequently, lowering the measured static water contact angle [32].

Table 5 shows a comparison between the two magnetic mixed matrix membranes: the magnetic mixed PES matrix from the previous work [24] and the magnetic mixed CA matrix membranes in this work. The blank PES W and CAP W membranes did not demonstrate oxygen permeation. Adding the magnetic alloys resulted in greater efficiency in the oxygen transmission: the magnetic mixed CA matrix membranes containing starfish-like Fe_10_Ni_90_ alloy showed 1.271 × 10^−5^ cm^3^/(m^2^·s) oxygen transmission and a high coercivity of up to 109 emu/g compared to 62.28 emu/g for the mixed CA matrix membranes containing necklace-like Fe_20_Ni_80_ alloy. The coercivity (Hc) levels of the blank CA (blank CAP WO: 12.21; blank CPA W: 19.89 emu/g) were much lower than those of the blank PES polymeric matrix (blank PES WO: 43.6; blank PES W: 79.3 emu/g). The addition of the same magnetic alloy/filler in the two polymeric matrices resulted in the oxygen transmission rate (OTR) of the magnetic mixed CAP1090 W membrane (1.271 × 10^−5^ cm^3^/(m^2^·s) being three times lower than the ORT of the magnetic mixed PES1090 W membrane (3.61 × 10^−5^ cm^3^/cm^2^·s). This could have been an indication of the syngeneic effect of the magnetic properties of the polymeric matrix and the added alloys/filler. This remark is supported by the close values for the coercivity, total porosity, and large-size porosity of both membranes (CAP1090 W and PES1090 W). The same was observed for the CAP2080 W and PES2080 W membranes. However, the coercivity of the blank membranes was higher than that of the magnetic CAP2080 W and PES2080 W membranes, which confirmed the greater efficiency of the starfish-like Fe_10_Ni_90_ alloy compared to the necklace-like Fi_20_Ni_80_ alloy.

Another important point was the compatibility between the polymeric matrix and the used alloys/fillers. The low OTR of the magnetic mixed CA matrix membranes compared to the magnetic mixed PES matrix membranes may be attributable to the lack of a gap between the added alloys/fillers and the used polymeric matrix; the total porosity of the magnetic mixed PES matrix membranes was slighter higher than the total porosity of the magnetic mixed CA matrix membranes. Consequently, we can confirm the compatibility between the polymeric matrix and the filler in sharing the oxygen transportation and, as a result, in the membrane productivity.

## 5. Conclusions

In this work, the oxygen transmission rate was found to be influenced by the affinity of the oxygen toward the magnetic fillers, which increased the transition rate of the oxygen in accordance with the increase in the concentration of the used filler. However, this affinity was different with different filler compositions and, accordingly, morphologies and magnetic properties. However, neither of the magnetic mixed CA matrix membranes showed significant differences in their determined total porosity.

Poly(methylmethacrylate) was blended with the CA in a minor percentage in order to improve the mechanical properties. Although lithium chloride was used in order to improve the porosity of the fabricated mixed CA matrix membranes, the total porosity of the fabricated membranes did not change. However, large-size pores were created in the sponge-supporting back layer. The compatibility between the polymeric matrix and the filler plays a decisive role in the membrane separation mechanism and, consequently, the membrane productivity.

The presented magnetic mixed CA matrix membranes open new areas for the use of mixed-matrix membranes in different applications and on an industrial scale. These membranes combine the advantages of both low-cost common polymers and low-cost, simply prepared inorganic fillers, making them more efficient and enabling them to be used in a wider range of applications without the need for an external magnetic field during either the membrane casting or the separation process.

## Figures and Tables

**Table 1:** Coded names and compositions of the fabricated magnetic mixed CA matrix membranes.

No.	Membrane Code	Filler Concen. (wt.%)	Filler Shape	LiCl Concen. (wt.%)	PMMA Concen. (wt.%)
1	Blank CA WO	0	No filler	0.0	0.0
2	Blank CAP WO	0	No filler	0.0	0.8
3	Blank CA W	0	No filler	0.1	0.0
4	Blank CAP W	0	No filler	0.1	0.8
5	CA1090 WO	2	Starfish-like	0.0	0.0
6	CAP1090 WO	2	Starfish-like	0.0	0.8
7	CA1090 W	2	Starfish-like	0.1	0.0
8	CAP1090 W	2	Starfish-like	0.1	0.8
9	CA2080 WO	2	Necklace-like	0.0	0.0
10	CAP2080 WO	2	Necklace-like	0.0	0.8
11	CA2080 W	2	Necklace-like	0.1	0.0
12	CAP2080 W	2	Necklace-like	0.1	0.8

**Table 2:** The monolayer adsorption amount (V_m_), adsorbate surface area (as), total pore volume, and average pore diameter of the prepared iron–nickel alloys used in this work.

Alloy Code	Adsorbed Amount (V_m_)	Surface Area (a_s_)	Total Pore Volume	Average Pore Diameter
	cm^3^ (STP) g^−1^	m^2^·g^−1^	cm^3^·g^−1^	nm
Fe_10_Ni_90_—starfish-like	1.0852	4.72	0.05	43.40
Fe_20_Ni_80_—necklace-like	1.7209	7.49	0.08	40.32

**Table 3:** EDX analysis of the fabricated blank CA and magnetic mixed CA matrix membranes using SEM equipment.

Membrane Code	Atomic %					Mass %				
	Fe	Ni	O	C	Cl	Fe	Ni	O	C	Cl
Blank CAP WO	0.00	0.00	52.87	47.13	0.00	0.00	0.00	59.91	40.09	0.00
Blank CA W	0.00	0.00	51.12	48.07	0.81	0.00	0.00	57.43	40.54	2.03
CAP1090 WO	0.16	0.75	39.42	59.67	0.00	0.64	3.15	45.04	51.17	0.00
CAP1090 W	0.35	0.64	50.22	48.74	0.05	1.35	2.59	55.50	40.44	0.12
CAP2080 WO	0.22	0.07	40.11	59.60	0.00	0.74	0.15	44.04	55.07	0.00
CAP2080 W	1.25	22.49	26.59	48.25	1.42	2.87	53.99	17.40	23.69	2.05

**Table 4:** EDX analysis of the fabricated blank CA and magnetic mixed CA matrix membranes using TEM equipment.

Membrane Code	Atomic %					Mass %				
	Fe	Ni	O	C	Cl	Fe	Ni	O	C	Cl
Blank CAP W	0.00	0.00	2.87	97.56	0.0	0.00	0.00	3.81	97.33	0.00
CAP1090 W	0.78	2.49	1.44	95.09	0.20	3.20	10.75	1.69	83.84	0.53
CAP2080 W	1.96	0.01	14.66	83.11	0.28	8.10	0.03	17.35	73.84	0.73

**Table 5:** Comparison between the magnetic mixed poly(ethersulfone) (PES) matrix [24] and the magnetic mixed cellulose acetate (CA)-matrix membranes regarding the coercivity (Hc), total and large-size porosity, and oxygen transmission rate (OTR).

No.	Membrane Code	Filler Concen. (wt.%)	Filler Shape	Coercivity (Hc) (emu/g)	Total Porosity (%)	Large-Size Porosity (%)	OTR cm^3^/(m^2^·s) × 10^−5^
1	Blank PES W	0	No filler	79.33	89 ± 1.0	70 ± 6	0.00
2	Blank CAP W	0	No filler	19.89	87 ± 2.0	82 ± 2	0.01
3	PES1090 W	2	Starfish-like	103.40	93 ± 0.7	88 ± 3	3.61
4	CAP1090 W	2	Starfish-like	109.26	91 ± 0.8	89 ± 1	1.27
5	PES2080 W	2	Necklace-like	73.67	92 ± 0.7	86 ± 2	1.36
6	CAP2080 W	2	Necklace-like	62.28	91 ± 2.0	84 ± 2	0.57

## Data Availability

All data generated or analysed during this study are included in this published article.

## References

[1] Aroon M.A., Ismail A.F., Matsuura T., Montazer-Rahmati M.M. (2010). Performance studies of mixed matrix membranes for gas separation: A review. Sep. Purif. Technol..

[2] Qadir D., Mukhtar H., Keong L.K. (2017). Mixed matrix membranes for water purification applications. Sep. Purif. Rev..

[3] Tavolaro P., Martino G., Andò S., Tavolaro A. (2016). Fabrication and evaluation of novel zeolite membranes to control the neoplastic activity and anti-tumoral drug treatments in human breast cancer cells. Part 1: Synthesis and characterization of pure zeolite membranes and mixed matrix membranes for adhesion and growth of cancer cells. Mater. Sci. Eng. C.

[4] Lin L., Wang A., Zhang L., Dong M., Zhang Y. (2012). Novel mixed matrix membranes for sulfur removal and for fuel cell applications. J. Power Sources.

[5] Jia Z., Wu G. (2016). Metal-organic frameworks based mixed matrix membranes for pervaporation. Microporous Mesoporous Mater..

[6] Himma N.F., Wardani A.K., Prasetya N., Aryanti PT P., Wenten I.G. (2019). Recent progress and challenges in membrane-based O_2_/N_2_ separation. Rev. Chem. Eng..

[7] Shakeel I., Hussain A., Farrukh S. (2019). Effect analysis of nickel ferrite (NiFe_2_O_4_) and titanium dioxide (TiO_2_) nanoparticles on CH_4_/CO_2_ gas permeation properties of cellulose acetate based mixed matrix membranes. J. Polym. Environ..

[8] De Mukherjee R.S. (2014). Adsorptive removal of phenolic compounds using cellulose acetate phthalate–alumina nanoparticle mixed matrix membrane. J. Hazard. Mater..

[9] Nasir R., Mukhtar H., Man Z., Mohshim D.F. (2013). Material Advancements in Fabrication of Mixed-Matrix Membranes. Chem. Eng. Technol..

[10] Dudek G., Turczyn R., Strzelewicz A., Rybak A., Krasowska M., Grzywna Z.J. (2012). Preparation and characterization of iron oxides–polymer composite membranes. Sep. Sci. Technol..

[11] Chong K., Lai S., Thiam H., Teoh H., Heng S. (2016). Recent progress of oxygen/nitrogen separation using membrane technology. J. Eng. Sci. Technol..

[12] Moghadassi A.R., Rajabi Z., Hosseini S.M., Mohammadi M. (2014). Fabrication and modification of cellulose acetate based mixed matrix membrane: Gas separation and physical properties. J. Ind. Eng. Chem..

[13] Nouri M., Marjani A. (2019). Surface modification of a cellulose acetate membrane using a nanocomposite suspension based on magnetic particles. Cellulose.

[14] Sanaeepur H., Nasernejad B., Kargari A. (2015). Cellulose acetate/nano-porous zeolite mixed matrix membrane for CO_2_ separation. Greenh. Gases Sci. Technol..

[15] Ahmad A.L., Jawad Z.A., Low S.C., Zein SH S. (2014). A cellulose acetate/multi-walled carbon nanotube mixed matrix membrane for CO_2_/N_2_ separation. J. Membr. Sci..

[16] Mubashir M., Yeong Y.F., Lau K.K., Chew T.L., Norwahyu J. (2018). Efficient CO_2_/N_2_ and CO_2_/CH_4_ separation using NH_2_-MIL-53 (Al)/cellulose acetate (CA) mixed matrix membranes. Sep. Purif. Technol..

[17] Sahraei R., Shahalizade T., Ghaemy M., Mahdavi H. (2018). Fabrication of cellulose acetate/Fe_3_O_4_@GO-APTS-poly(AMPS-co-MA) mixed matrix membrane and its evaluation on anionic dyes removal. Cellulose.

[18] Chaudhary M., Maiti A. (2020). Fe–Al–Mn@ chitosan based metal oxides blended cellulose acetate mixed matrix membrane for fluoride decontamination from water: Removal mechanisms and antibacterial behavior. J. Membr. Sci..

[19] De Guzman M.R., Andra CK A., Ang MB M.Y., Dizon GV C., Caparanga A.R., Huang S.-H., Lee K. (2021). Increased performance and antifouling of mixed-matrix membranes of cellulose acetate with hydrophilic nanoparticles of polydopamine-sulfobetaine methacrylate for oil-water separation. J. Membr. Sci..

[20] Abdel-Karim A., El-Naggar M.E., Radwan E.K., Mohamed I.M., Azaam M., Kenawy E.-R. (2021). High-performance mixed-matrix membranes enabled by organically/inorganic modified montmorillonite for the treatment of hazardous textile wastewater. Chem. Eng. J..

[21] Ali AS M., Soliman M.M., Kandil S.H., Khalil MM A. (2021). Emerging mixed matrix membranes based on zeolite nanoparticles and cellulose acetate for water desalination. Cellulose.

[22] Andrade M.C., Pereira J.C., de Almeida N., Marques P., Faria M., Gonçalves M.C. (2021). Improving hydraulic permeability, mechanical properties, and chemical functionality of cellulose acetate-based membranes by co-polymerization with tetraethyl orthosilicate and 3-(aminopropyl)triethoxysilane. Carbohydr. Polym..

[23] Nady N., Salem N., Mohamed M.A., Kandil S.H. (2021). Iron-Nickel Alloy with Starfish-like Shape and Its Unique Magnetic Properties: Effect of Reaction Volume and Metal Concentration on the Synthesized Alloy. Nanomaterials.

[24] Nady N., Salem N., Kandil S.H. (2022). Novel magnetic iron–nickel/poly(ethersulfone) mixed matrix membranes for oxygen separation potential without applying an external magnetic field. Sci. Rep..

[25] Abdel-Karim A., Leaper S., Alberto M., Vijayaraghavan A., Fan X., Holmes S.M., Souaya E.R., Badawy M.I., Gorgojo P. (2018). High flux and fouling resistant flat sheet polyethersulfone membranes incorporated with graphene oxide for ultrafiltration applications. Chem. Eng. J..

[26] Frommer M.A., Messalen R.M. (1973). Mechanism of membrane formation, VI. Convective flows and large voids formation during membrane preparation. Ind. Eng. Chem. Prod. Res. Dev..

[27] Cheng Y.-T., Rodak D.E., Angelopoulos A., Gacek T. (2005). Microscopic observations of condensation of water on lotus leaves. Appl. Phys. Lett..

[28] Endoh R., Tanaka T., Kurihara M., Ikeda K. (1977). New Polymeric materials for reverse osmosis membranes. Desalination.

[29] Mohd Shafie ZM H., Ahmad A.L., Low S.C., Rode S., Belaissaoui B. (2020). Lithium chloride (LiCl)-modified polyethersulfone (PES) substrate surface pore architectures on thin poly(dimethylsiloxane) (PDMS) dense layer formation and the composite membrane’s performance in gas separation. RSC Adv..

[30] Ali U., Karim K.J.B.A., Buang N.A.A. (2015). review of the properties and applications of poly(methyl methacrylate)(PMMA). Polym. Rev..

[31] Ito A., Shin A., Nitta K. (2022). Additive effects of lithium halides on the tensile and rheological properties of poly(methyl methacrylate). Polym. J..

[32] Sudiarti T., Wahyuningrum D., Bundjali B., Made Arcana I. (2017). Mechanical strength and ionic conductivity of polymer electrolyte membranes prepared from cellulose acetate-lithium perchlorate. IOP Conf. Ser. Mater. Sci. Eng..

